# Psychometric Properties of the Spanish Version of the Cognitive Emotional Regulation Difficulties Questionnaire (CERQ) in Higher Education Students in Times of Covid-19

**DOI:** 10.3389/fpsyg.2021.695147

**Published:** 2021-06-04

**Authors:** Clemente Rodríguez-Sabiote, Pilar Ibáñez-Cubillas, Slava López-Rodríguez, José Álvarez-Rodríguez

**Affiliations:** ^1^Departament of Research Methods and Diagnosis in Education, Faculty of Education Sciences, University of Granada, Granada, Spain; ^2^Department of Education Sciences, Faculty of Education, University of Extremadura, Badajoz, Spain; ^3^Department of Didactics of Language and Literature, Faculty of Education Sciences, University of Granada, Granada, Spain; ^4^Department of Pedagogy, Faculty of Education Sciences, University of Granada, Granada, Spain

**Keywords:** emotion regulation, psychometric property, higher education, pandemic Covid-19, students

## Abstract

The Cognitive Emotion Regulation Questionnaire (CERQ) is an instrument developed to assess the cognitive strategies of emotional regulation used by people after experiencing a negative event. The present study aimed to validate the Spanish version of the CERQ in students of the University of Granada (Spain) during the Covid-19 homebound. An online scale was developed and applied based on the Spanish version of the CERQ-S36, consisting of 36 items structured around nine cognitive strategies. Using a mixed sampling, the scale was applied to 450 students from different degree programmes. Regarding the psychometric results of the scale, firstly, in relation to reliability as internal consistency, we found that the scale applied to University students in a Covid-19 setting is highly stable. Secondly, in reference to concurrent criterion validity, we can conclude that the items individually measure the same as the total scale (taken as internal criterion), and thirdly and finally, in relation to construct validation, the two factor analyses implemented, one exploratory and the other confirmatory in nature, conform a factor structure of latent dimensions identical to the original one. In conclusion, the results obtained as a whole suggest that the CERQ-S36 scale could be useful for assessing cognitive coping in University populations in times of crisis. In situations such as the current global emergency due to the presence of Covid-19, the scale is useful for understanding emotional regulation strategies. More studies should be carried out with this scale to find out how emotions influence and what consequences they have on the health and psychological functioning of University students in times of social crisis.

## Introduction

The SARS-Cov-2 coronavirus pandemic and the disease it causes (covid-19) is affecting countries all over the world. Due to its rapid spread, governments have adopted various containment measures. In the case of Spain, a state of alarm was declared and quarantine was established throughout the national territory from March to June 2020. In addition, a series of extraordinary measures have been applied, such as social distancing or the extreme limitation of contacts and group activities, which is currently in force. The consequences of the pandemic (e.g., deaths, interruption of mourning rituals, economic paralysis, losses, or possible financial ruin), the imposed restriction measures or the risk of contagion are causing a major change in people's lives. Indeed, such a disruptive event has a strong impact on the mental health of the population, resulting in psychosocial disturbances (restlessness, uncertainty, insecurity, social disorder) (Scholten et al., 2020), psychological and emotional desorders (depression, irritability, stress, anxiety, insomnia, fear, confusion, anger, frustration, boredom, post-traumatic symptoms) (Brooks et al., 2020). Recent work identifies that emotional regulation through thought or cognition is related to psychological health (Gross, 2015; Potthoff et al., 2016), demonstrating that emotions and affective states influence a person's well-being and health (Sloan et al., 2017). In this framework, it is important to recognise that people face complex and stressful situations, which challenge the mechanisms of emotional regulation and adaptive capacity to maintain an optimal state of health.

Emotions and their expression are part of everyday life and allow people to adapt to different situations. They act as an alarm system, indicating aversive, dangerous or pleasant events and generate an adaptive reaction to respond to the environment (Garnefski et al., 2002a). Emotions emerge in a situation that is significant for the person. These involve a series of physiological, behavioural and psychological changes, which require an interpretation of the situation in order that the individual performs a certain action and communicates his or her emotional state. It is assumed that emotions emerge from an assessment and interpretation of the situation. Therefore, the same situation can provoke different emotions in different people. Hence, the intensity of the emotional experience depends on the significance of the event that generates it (Reeve, 2005; Gross and Thompson, 2007).

According to Gómez-Pérez and Calleja-Bello (2017, p. 98), “emotion is an individual's response to environmental stimuli that coordinates different systems and aims to provide information to influence him/her according to his/her needs.” Emotional response is composed of a set of systems that influence personal experience, expression, physiological, and behavioural responses (Gómez-Pérez and Calleja-Bello, 2017). In fact, Reeve (2005) indicates that emotion is composed of four dimensions or components: feeling (gives meaning to the emotion by providing subjectivity to the experience), body activation (consists of the activation of the biological system that prepares and regulates the body's adaptive behaviour during the emotion), purpose (is an intentional aspect that generates an impulse to action, which explains people's actions during the emotion), and expressive behaviour (is the communicative aspect of the emotion, such as posture, gestures or facial expressions). In this sense, emotion is understood as a multidimensional process (Gross and Thompson, 2007), as it involves a series of phenomena that appear coordinated and simultaneously in the process of emotional reaction and its regulation (Reeve, 2005).

Emotions guide behaviours in various situations (Reeve, 2005) and emotion regulation modifies the emotional experience by consciously controlling certain elements. Thus, a lack of negative emotion regulation can be problematic and interfere with a person's life when the change in emotional response is undesirable, intense or long-term (Gross and Thompson, 2007). Because emotions and their regulation largely determine people's behaviour, their study has gained great importance, becoming one of the psychological variables with the greatest impact (Gómez-Pérez and Calleja-Bello, 2017).

Emotional regulation is defined as “any strategy aimed at maintaining, increasing or suppressing an ongoing affective state,” including the ability to regulate physiological changes linked to the emotion in order to provide an appropriate response to the context (Thompson, 1994, p. 27). Emotional regulation involves the activation of those mechanisms aimed at controlling, evaluating and modifying emotional reactions, in order to redirect the flow of positive or negative emotions, their intensity and duration (Gross, 1998; Koole, 2009).

Initially, Gross and John (2003) indicated that emotions arise in a four-step process: relevant situation, attention, evaluation, and emotional response. However, in a more recent study, Gross (2015) shows in his extended model of emotion regulation that both emotion generation and emotion regulation involve a cyclical appraisal system based on three stages: identification (deciding whether to regulate or alter an emotion), selection (choosing the regulation strategy to be used) and implementation (implementing the chosen strategy).

Although there are different ways of regulating emotions (Pascual Jimeno and Conejero López, 2019). Garnefski and Kraaij (2007) indicate that the cognitive processes involved in emotional experience play a key role. The authors propose the existence of nine strategies for regulating emotions based on cognitive processes: (1) Blaming others, a process in which the thoughts of guilt generated by the experienced situation fall on others; (2) Self-blaming, an individual blames oneself for the experienced situation; (3) Obsessive reflection, focusing systematically and excessively on the negative feelings and thoughts associated with the event; (4) Catastrophism, the negative emotions and thoughts linked to the event are emphasised and magnified; (5) Putting into perspective, an attempt is made to diminish the seriousness of the situation by relativising it and comparing it with other events; (6) Positive reinterpretation, which consists of generating thoughts that give a positive meaning to the event in terms of personal growth; (7) Focus on plans, which consists of thinking about the steps to follow to manage the negative event; (8) Positive focus, which consists of generating pleasant and cheerful thoughts instead of focusing on the problematic situation; and (9) Acceptance, which consists of accepting the reality, the negative event and the thoughts, feelings, or sensations generated. The first four strategies indicate maladaptive emotion regulation and the last five would be adaptive.

The scientific literature shows that when people are not able to regulate their emotions effectively or use maladaptive strategies, emotional regulation difficulties can lead to various forms of pathology (Garnefski et al., 2002a; Sheppes et al., 2015; Domínguez-Lara, 2017) such as anxiety (Domínguez-Lara, 2017; Del Valle et al., 2018), depression (Joormann and Stanton, 2016; Domínguez-Lara, 2017), or stress (González et al., 2017). In this sense, emotional regulation has been studied and linked to mental and physical well-being (Sloan et al., 2017), being incorporated as an explanatory variable in psychopathology. Other topics linked to emotional regulation have also been studied, for example, the benefit of mindfulness-based intervention (Brockman et al., 2017; Guendelman et al., 2017; Iani et al., 2019), emotional regulation as a predictor of substance abuse and addictive behaviours (Tang et al., 2016; Estevez et al., 2017), the use of maladaptive strategies in eating disorders (Dingemans et al., 2017; Goldschmidt et al., 2017), emotional regulation in children and adults with Autism Spectrum Disorder (Bruggink et al., 2016; Berkovits et al., 2017), emotional regulation according to culture (Potthoff et al., 2016) or its relationship with emotional intelligence (Peña-Sarrionandia et al., 2019).

The Cognitive Emotional Regulation Questionnaire (CERQ) was developed by Garnefski et al. (2002b) to assess cognitive strategies of emotional regulation. The instrument consists of a 36-item scale that explores the nine cognitive strategies (blaming others, self-blame, obsessive reflection, catastrophizing, putting into perspective, positive reinterpretation, focus on plans, positive focus, and acceptance) of Garnefski et al. (2002a) model of emotional regulation.

Originally, the CERQ was designed in the Netherlands and used with adults and adolescents (Garnefski et al., 2002b). Later, a version for children aged 9–11 years was developed (Garnefski et al., 2007). The questionnaire has been validated and adapted to different countries and populations; Romania, with participants aged 13–18 and 18–67 years (Perte and Miclea, 2011); Hungary, in undergraduate and graduate students (Miklósi et al., 2011); Turkey, with participants aged 18–47 years (Tuna and Bozo, 2012); Iran, with University students aged 18+ years (Abdi et al., 2012); China, in children aged 9–11 years and University students aged 17–26 years (Zhu et al., 2008; Liu et al., 2016); Brazil, with a sample with an average age of 22.7 years (Schäfer et al., 2018); Argentina, in University students with an average age of 24.6 years (Medrano et al., 2013); France, with participants aged 18–37 years (Jermann et al., 2006); Italy, participants aged 20–87 years (Balzarotti et al., 2016); Portugal, participants aged 18–60 years (Martins et al., 2016) or Germany, validated in clinical population (Görgen et al., 2015).

In Spain, there are three versions of the CERQ: the CERQ-S validated in a sample aged between 16 and 58 years and with Cronbach's α values between 0.60 and 0.89 in the different dimensions (Domínguez-Sánchez et al., 2011); the validated version of the CERQ-SA in adolescents (sample aged 14–18 years) which exhibits reliability values α = 0.89 (Chamizo-Nieto et al., 2020) and the CERQ-Sk, for children aged 7–12 years which shows an overall reliability of α = 0.88 (Orgilés et al., 2018).

As shown, the CERQ is a versatile instrument that allows its application in different languages, population sectors and contexts. Although there are other instruments that assess processes linked to emotional regulation, such as the Psychological Well-Being Scale for Adults (BIEPSA; Casullo, 2002), the Emotional Fatigue Scale (ECE; Fontana, 2011), the Difficulties in Emotion Regulation Scale (DERS; Gratz and Roemer, 2004) or the Behavioural Emotion Regulation Questionnaire (BERQ- Kraaij and Garnefski, 2019), the CERQ “is the only questionnaire that assesses cognitive strategies of emotion regulation” (Medrano et al., 2013, p. 86) and allows us to understand its relationship with emotional problems. We have recently used it in a comprehensive research study (Fernández Cruz et al., 2020) from which we extract the data shown in this study.

## Methods

### Participants

The questionnaire was applied via a convenience and snowball sampling (Kalton, 2020) and distributed through different networks of University professors who, interested in the study, were willing to send it to their students requesting their collaboration. The number of participating students from the University of Granada was 450, since this research is incardinated within a study with a larger sample size of several countries with overall results (Fernández Cruz et al., 2020).

By disciplines, 68.2% correspond to Social and Legal Sciences (students of Primary Education, Infant Education, Economics, Pedagogy degrees, etc.), 9.8% to Sciences, specifically the Degree in Biology, 7.2% to Health Sciences (Nursing and Medicine Degrees), 7.9% to Engineering and Architecture (Architecture Degree), and finally, the remaining 6.9% to the Degree and Humanities (specifically the Degrees in English Philology and Philosophy). By gender, 80% are women and the remaining 20% are men. Finally, by age: minimum age = 18; maximun years = 37 with a mean = 22.55 years and a standard deviation = 5.77 years.

### Instrument

For the collection of information and validation of the scale we used an original scale called Cognitive Emotional Regulation Questionnaire (CERQ-36), developed by Garnefski et al. (2002a) and Garnefski and Kraaij (2007), although we have finally adopted the Spanish version CERQ-s36 (long version) by Domínguez-Sánchez et al. (2011). This scale consists of 36 items presented in Likert format with five response options ranging from almost never (1) to nearly always (5). In turn, each of these 36 items is structured around nine cognitive strategies, namely: Rumination (items 3, 12, 21, and 30), Catastrophizing (items 8, 17, 26, and 35), Self-blame (items 1, 10, 19, and 28), and Other-blame (items 9, 18, 27, and 36). These first four strategies or factors would make up a meta-factor that we shall call less adaptive strategies to an emergency situation. On the other hand, we also find the strategy Putting perspectives (items 7, 16, 25, and 34), Acceptance (items 2, 11, 20, and 29), Positive refocusing (4, 13, 22, and 31), Positive reappraisal (6, 15, 24, and 33), and Refocus in Planning (5, 14, 23, and 32). These five strategies or factors, on the contrary, would form a meta-factor that we could call more adaptive strategies towards an emergency situation.

### Research Design, Administration Procedure, and Scale Data Analysis

From the methodological point of view, the proposed research design obeys to an instrumental type of research (Shaughnessy et al., 2000) or also called test validation design (Crocker and Algina, 1986) consisting of the calculation of the quality parameters contemplated by the Classical Test Theory (CTT). The application of the scale was carried out online during the period from 14 March 2020 (the day the Covid-19 alert entered into force throughout Spain) till 15 April 2020. For this purpose, an online scale was developed using google forms (google survey) based on the adaptation of the Spanish version of the CERQ scale by Domínguez-Sánchez et al. (2011). Participation was voluntary and all participants were informed about the purposes of the research and the anonymous and confidential nature of their responses, prior to obtaining their consent. For data analysis we used together Factor (v.10, Ferrando and Lorenzo-Seva, 2017), JAMOVI (v.1.6.16, The jamovi project, 2020), Stata (v.15, StataCorp, 2017) and SPSS (v.26, IBM Corp, 2019), implementing, in addition to the relevant analyses on the internal consistency of the scale, the concurrent criterion validity, as well as the appropriate analyses to determine the structure of the scale (construct validity) by means of exploratory and confirmatory factor analyses.

## Results

### Reliability and Criterial Validity of Scale

#### Internal Consistency of Scale

First, we present the reliability results of the instrument based on its internal consistency, as we only have one application of the instrument. The omega ordinal internal consistency coefficient is conceptually similar to Cronbach's α. The main difference is that the omega is based on the polychoric correlation matrix between items (more suitable for ordinal data) as in our case, rather than on Pearson's covariance (correlation) matrix (more suitable for continuous data). For that reason, we have decided to consider only the McDonald's ω more appropriate for estimating internal consistency when the data are ordinal (Gadermann et al., 2012).

The McDonald's ω value is 0.83. The result obtained confirm a high internal consistency of the scale (McDonald, 1999; Katz, 2006). Given that the former is more robust (Viladrich et al., 2017) than the latter, we can affirm that possible biases, due to uncorrelated errors or to the tau-equivalence measurement model, are duly neutralised (Dunn et al., 2014). Regarding the results of McDonald's ω coefficients by factors, firstly, we have a value of 0.71 for Self-blame, a value of 0.72 for Acceptance; 0.81 for Rumination; 0.92 for Positive Reapprasial; 0.73 for Refocus Planning; 0.86 for Positive Refocusing; 0.72 for Putting Perspective; 0.76 for Catastrophizing, and finally 0.94 for Other blame. As can be seen, in general, McDonald's ω moderately high values have been obtained, reporting good internal consistency, not only for the scale as a whole, but also broken down by factors (Zumbo et al., 2007).

In addition, we calculated the composite reliability, a coefficient very similar to McDonald's ω, but with the advantage that it takes into account the intercorrelation established between the latent factors extracted and the average variance extracted (AVE) from each factor using the JAMOVI program (v.1.6.16, The jamovi project, 2020) and Excel spreadsheet. For this purpose, we have taken into consideration the standardised factor loadings of each item in reference to the latent factor of which it is a part, as well as the residual covariances associated with the resulting standardised factor loadings.

The results obtained are shown in the Table 1.

**Table 1:** Composite reliability and average variance extracted values of each factor.

**Factor**	**Composite reliability**	**AVE**
Self-blame	0.701	0.334
Acceptation	0.721	0.513
Rumiation	0.798	0.502
Positive- reappraisal	0.901	0.706
Refocus in planning	0.745	0.521
Positive refocusing	0.849	0.567
Putting perspectives	0.789	0.473
Catastrophizing	0.778	0.472
Other blame	0.910	0.734

Composite reliability values ≥0.70 are preferable (Hair et al., 2018), but up to 0.60 are acceptable (Bagozzi and Yi, 1988). As can be seen, all the factors have obtained composite realibility values >0.70 which, in the case of some factors, the fewest of course, are close to unity (1). On the other hand, we can also see that the average variances extracts (AVE's) range from AVE = 0.334 for the Self-blame factor to AVE = 0.734 for the Other blame factor. Given that the average variances extracts show the ratio between the variance of each factor j in relation to the total variance due to the measurement error of that factor and that values of average variances extracted (AVE ≥0.50) are advisable (Fornell and Larcker, 1981 and Nunnally and Bernstein, 1994), we can affirm that adequate results have been achieved in this respect in most of the factors, except for the Self-blame, Item reliability and criterial validity statistics.

To determine the concurrent criterion validity of the scale, we calculated the item-rest correlation, i.e., the correlation between each item and the total scale. In most of the cases reported, *r* > 0.35 was obtained, which seems to indicate that the items individually measure the same as the total scale (internal criterion). On the other hand, McDonald's ω coefficient obtained for the if each item is dropped scale yield values that are never higher than those obtained for the scale as a whole. This is an indicator showing that the items that make up the scale are not dispensable, given that the internal consistency after their elimination would not improve.

## Factor Structure of Scale

Before investigating the stability of the factor structure of the CERQ scale at a time of global emergency such as that of Covid-19, a descriptive analysis of the items was carried out, focusing on compliance with the assumption of normality, both univariate and multivariate. For this purpose, descriptive measures of mean, standard deviation, skewness and univariate kurtosis were calculated, as well as various multivariate normality tests.

As can be seen, some of the items considered have obtained values of skewness and kurtosis that are certainly high (for example, items 1, 8, 10, etc.), although, on the contrary, most of them have apparently achieved values in both dimensions that could indicate the presence of univariate normality. If we take into account the considerations of Curran et al. (1996) that for univariate normality to be fulfilled, values of the coefficient of skewness between (−2.2) and of kurtosis between (−7.7) should be obtained, we could consider that a large number of the items that make up the scale conform to univariate normality (Table 2).

**Table 2:** Univariate descriptives and test for univariate normality.

**Item**	**Mean**	**Sd**.	**Skewness**	**Kurtosis**	Skewness criterion [−2,2]^*^	Kurtosis criterion[−7,7]^*^
1	1.12	0.468	4.901	27.039	No	No
2	3.74	1.239	−0.641	−0.736	Yes	Yes
3	3.38	1.313	−0.344	−1.098	Yes	Yes
4	2.88	1.252	0.238	−1.023	Yes	Yes
5	3.39	1.217	−0.262	−0.950	Yes	Yes
6	3.74	1.227	−0.654	−0.676	Yes	Yes
7	2.89	1.295	0.230	−1.102	Yes	Yes
8	1.56	0.990	1.904	2.906	No	No
9	2.72	1.405	0.352	−1.187	Yes	Yes
10	1.25	0.655	3.329	12.704	No	No
11	3.82	1.186	−0.687	−0.518	Yes	Yes
12	3.12	1.318	−0.087	−1.119	Yes	Yes
13	2.84	1.210	0.229	−0.965	Yes	Yes
14	3.32	1.169	−0.183	−0.915	Yes	Yes
15	3.25	1.313	−0.114	−1.250	Yes	Yes
16	4.21	0.994	−1.203	0.605	Yes	Yes
17	2.64	1.257	0.355	−0.941	Yes	Yes
18	2.82	1.464	0.286	−1.321	Yes	Yes
19	1.85	0.965	1.115	0.745	Yes	Yes
20	2.45	1.315	0.635	−0.743	Yes	Yes
21	2.73	1.341	0.248	−1.149	Yes	Yes
22	2.90	1.253	0.239	−1.029	Yes	Yes
23	2.79	1.200	0.268	−0.910	Yes	Yes
24	2.76	1.337	0.325	−1.090	Yes	Yes
25	2.81	1.347	0.264	−1.138	Yes	Yes
26	1.84	1.085	1.342	1.101	Yes	Yes
27	2.76	1.234	0.384	−0.887	Yes	Yes
28	1.35	0.777	2.623	7.308	No	No
29	3.55	1.110	−0.223	−0.843	Yes	Yes
30	3.16	1.221	−0.138	−0.980	Yes	Yes
31	2.94	1.226	0.141	−1.013	Yes	Yes
32	2.67	1.190	0.331	−0.830	Yes	Yes
33	2.93	1.248	0.184	−1.007	Yes	Yes
34	3.28	1.312	−0.075	−1.256	Yes	Yes
35	2.58	1.292	0.494	−0.844	Yes	Yes
36	2.49	1.455	0.599	−1.032	Yes	Yes

* *Skewness and kurtosis criterion based on Curran et al. (1996)*.

On the other hand, to test for multivariate normality we calculated Mardia's coefficients (Mardia, 1970) for mSkewness = 207.51 and mKurtosis = 1508.53, as well as the Henze-Zikler coefficient = 1.019. In all three cases (see Table 3) the results point to the violation of multivariate normality, given that the χ^2^ values obtained are associated with significance levels *p* < 0.05.

**Table 3:** Test for multivariate normality.

**Test**	**Chi^**2**^ value**	**Sig**.
Mardia mSkewness = 207.5101	chi^2^(8436) = 14565.929	Prob>chi^2^ = 0.000^***^
Mardia mKurtosis = 1508.533	chi^2^(1) = 754.320	Prob>chi^2^ = 0.000^***^
Henze-Zirkler = 1.019653	chi^2^(1) = 2.36e+07	Prob>chi^2^ = 0.000^***^

* *p < 0.05*,

** *p < 0.01*,

*** *p < 0.001*.

We consider that the best method to perform a CFA is the Maximum Likelihood (ML^1^) method in which, although the presence of univariate and multivariate normality is necessary (Hox et al., 2010), there is evidence that the ML estimation method is an adequate method to obtain factor loadings, even if the assumption of multivariate normality is not met (Beaducel and Herberg, 2006).

### Exploratory Factor Analysis

After checking the univariate and multivariate normality assumptions, we decided to apply an exploratory factor analysis. The characteristics of this exploratory factor analysis are based on *the principal component extraction method with Kaiser's criterion (*λ ≥ *1) and the rotation considered: varimax*. With respect to assumption cheques of adequacy of the Pearson correlation matrix we highlight that the determinant of the matrix is aproximately I*A*I = 0.000001, while Bartlett's test of Sphericity = 4664.7 (df = 630, *p* = 0.000010) and the Kaiser-Meyer-Olkin (KMO) test = 0.865 (BC Bootstrap 95% confidence interval of KMO = 0.865–0.866), as well as measures of sampling adequacy (MSA) with minimum values, MSA >0.70, up to values that in some items reach MSA = 0.92. All these results point to the convenience of carrying out exploratory factor analysis given that, firstly, the determinant of the resulting matrix is close to 0, without reaching this value, which can be considered that we are not dealing with a singular matrix and that the variables as a whole are not linearly dependent. Secondly, and not less important, the values of the measures of sampling adequacy overall (KMO) and that of each of the items (MSA) can be considered as fairly good and, in any case, they indicate that the direct correlations between pairs of items are more important than the partial correlations. Thirdly, the Bartlett's test of Sphericity is associated with a *p* < 0.001, which indicates that we are not dealing with an identity matrix characterised by the presence of perfect correlations on the diagonal and null correlations in the rest and that, therefore, there are empirical indications of the presence of intercorrelated items. The results obtained in relation to the factor loadings after the relevant rotation and elimination of those with *r* < 0.35, as well as the eigenvalues, variances explained by each factor, and their denomination are shown below.

From the resulting factor solution shown in the Table 4, we first highlight the variances obtained for each of the empirical variables observed (the 36 items). In turn, this variance of each variable is broken down into two sources. On the one hand, that which depends on the common factors (communality) and, on the other hand, that which depends on the specific factor or measurement error (uniqueness). In almost all items, the communality is higher than the uniqueness, which means that the proportion of variance of each item “j” explained by the common factors is higher than that due to the error and that, therefore, almost all items are adequately represented in the resulting factor solution. In reference to the interpretation, we have obtained a factor model that confirms the presence of the 9 classical components of the CERQ scale with a total explained variance of almost 64%. In our particular case and in order of importance, the following dimensions appear: Positive Reappraisal (19.5% explained variance and λ1 = 7.03), Rumination (14.5% explained variance and λ2 = 5.19), Other Blame (8% explained variance and λ3 = 2.89), Positive Refocusing (4.9% explained variance and λ4 = 1.77), Putting Perspectives (4.8% explained variance and λ5 = 1.75), Self-Blame (4% explained variance and λ6 = 1.46), Acceptance (3.7% explained variance and λ7 = 1.36), Catastrophizing (2.9% explained variance and λ8 = 1.05), and Refocus in Planning (2.5% explained variance and λ9 = 0.91).

**Table 4:** Exploratory factor analysis by principal component analysis and explained variance based on eigenvalues.

	**Component Loadings**									
	**Component**									**Uniqueness (Ψ=1–h^**2**^) /Communality (h^**2**^)**
	**1**	**2**	**3**	**4**	**5**	**6**	**7**	**8**	**9**	
i1						0.765				0.370/0.630
i2							0.868			0.208/0.792
i3		0.742								0.418/0.582
i4	0.788									0.264/0.736
i5									0.602	0.422/0.578
i6				0.721						0.394/0.606
i7					0.542					0.629/0.371
i8								0.701		0.361/0.639
i9			0.895							0.195/0.805
i10						0.753				0.403/0.597
i11							0.876			0.179/0.821
i12		0.697								0.412/0.588
i13	0.844									0.202/0.798
i14									0.368	0.461/0.539
i15				0.769						0.289/0.711
i16					0.588					0.456/0.544
i17								0.431		0.408/0.592
i18			0.939							0.110/0.890
i19						0.595				0.525/0.475
i20							0.680			0.327/0.673
i21		0.674								0.439/0.561
i22	0.798									0.225/0.775
i23									0.686	0.362/0.638
i24				0.716						0.310/0.690
i25					0.769					0.298/0.702
i26							0.456			0.441/0.559
i27			0.754							0.344/0.656
i28						0.667				0.519/0.481
i29							0.533			0.474/0.526
i30		0.782								0.339/0.661
i31	0.812									0.244/0.756
i32									0.412	0.469/0.531
i33				0.740						0.270/0.730
i34					0.718					0.392/0.608
i35		0.550						0.357		0.372/0.628
i36			0.901							0.166/0.834
λ (Eigenvalue)	7.03	5.19	2.89	1.77	1.75	1.46	1.36	1.05	0.911	-
Proportionvariance	0.195	0.144	0.080	0.049	0.048	0.040	0.037	0.029	0.025	-
CumulativeProportion and % of variance	0.195 19.5%	0.3333%	0.42 42%	0.45945.9%	0.507 50.7%	0.54754.7%	0.584 58.4%	0.61361.3%	0.638 63.8%	-
Factor name	PosReap.	Rumiat	Other-blame	PosRef	PutPers	Self-blame	Accept.	Catastr.	RefPlan	-

“*Varimax” rotation was used and deleted items with factor loadings r < 0.0.35*.

### Confirmatory Factor Analysis

For the development of the Confirmatory Factor Analysis we used the STATA v.15 programme using the ML estimation method. The main results obtained are grouped around the resulting path analysis and refer to the standardised parameters, specifically the different correlations between the different factors, as well as the standardised regression weights (factor loadings) of each exogenous latent variable or factor with the endogenous empirical variables that make it up, as well as, the standardised measurement errors. With regard to the various standardised regression weights, we should point out that they range from *r* = 0.16 (referring to the acceptation factor with item 20) to *r* = 0.96 (referring to the other blame factor with item 18). On the other hand, all the regression weights except the one referring to the acceptation factor with item 20 have obtained standardised scores *z* > 1.96 associated with statistical significance *p* < 0.001. As can be seen, we have obtained a factor structure identical to the original one of Garnefski et al. (2002b), made up of 9 factors of 4 items each. In order to appreciate the results obtained in their entirety, we offer the resulting standardized factor loadings (Table 5).

**Table 5:** Standardized factor loadings.

	***Measurement***	***Standardized coef*.**	***Std error***	***z***	***P > |z|***
p1	Self-blame	0.695363	0.0424057	16.40	0.000^***^
	cons	2.39110	0.0960797	24.89	0.000^***^
p10	Self-blame	0.619778	0.0427683	14.43	0.000^***^
	cons	1.91501	0.0823349	23.26	0.000^***^
p19	Self-blame	0.564876	0.0471911	11.97	0.000^***^
	cons	1.91646	0.0823753	23.27	0.000^***^
p28	Self-blame	0.543836	0.0458814	11.85	0.000^***^
	cons	1.74531	0.0776920	22.46	0.000^***^
p2	Accept	0.764230	0283179	26.99	0.000^***^
	cons	3.02054	0.1153510	26.19	0.000^***^
p11	Accept	0.886848	0.0268939	32.98	0.000^***^
	cons	3.22048	0.1216491	26.47	0.000^***^
p20	Accept	0.159077	0.0530666	0.308	0.764
	cons	1.86639	0.0809886	23.05	0.000^***^
p29	Accept	0.559574	0.0393980	14.20	0.000^***^
	cons	3.20578	0.1211838	26.45	0.000^***^
p3	Rumiation	0.6916535	0.0319787	21.63	0.000^***^
	cons	2.579708	0.1017485	25.35	0.000^***^
p12	Rumiation	0.6778654	0.0328664	20.62	0.000^***^
	cons	2.370937	0.0954889	24.83	0.000^***^
p21	Rumiation	0.6756703	0.0328918	20.54	0.000^***^
	cons	0.0859088	0.0224613	23.77	0.000^***^
p30	Rumiation	0.7561679	0.0283881	26.64	0.000^***^
	cons	2.58963	0.1020494	25.38	0.000^***^
p4	PositReap	0.8715127	0.0205546	38.96	0.000^***^
	cons	2.305989	0.0935585	24.65	0.000^***^
p13	PositReap	0.8715127	0.0154198	56.52	0.000^***^
	cons	2.350256	0.0948648	24.77	0.000^***^
p22	PositReap	0.8600975	0.0162454	52.95	0.000^***^
	cons	2.315132	0.0938277	24.67	0.000^***^
p31	PositReap	0.8119917	0.0196546	41.31	0.000^***^
	cons	2.403334	0.0964445	24.92	0.000^***^
p5	RefPlan	0.5910514	0.0385916	15.32	0.000^***^
	cons	2.792198	0.1082504	25.79	0.000^***^
p14	RefPlan	0.6946699	0.0330359	21.03	0.000^***^
	cons	2.838686	0.1096871	25.88	0.000^***^
p23	RefPlan	0.4812766	0.0437819	10.99	0.000^***^
	cons	2.331676	0.0943164	24.72	0.000^***^
p32	RefPlan	0.6794758	0.033122	20.51	0.000^***^
	cons	2.243751	0.0917299	24.46	0.000^***^
p6	PostRef	0.6796691	0.0316256	21.49	0.000^***^
	cons	3.05236	0.1163485	26.23	0.000^***^
p15	PostRef	0.7299764	0.0288017	25.34	0.000^***^
	cons	2.479593	0.0987262	25.12	0.000^***^
p24	PostRef	0.7353982	0.0276718	26.58	0.000^***^
	cons	2.068972	0.0866766	23.87	0.000^***^
p33	PostRef	0.8235364	0.0226975	36.28	0.000^***^
	cons	2.351958	0.0949169	24.78	0.000^***^
p7	PuttPers	0.4855183	0.0454255	10.69	0.000^***^
	cons	2.231816	0.0913813	24.42	0.000^***^
p16	PuttPers	0.5702385	0415828	13.71	0.000^***^
	cons	4.243226	0.1546912	27.43	0.000^***^
p25	PuttPers	0.6751473	0.037281	18.11	0.000^***^
	cons	2.087475	0.0872051	23.94	0.000^***^
p34	PuttPers	0.715837	0.0354726	20.18	0.000^***^
	cons	2.501083	0.0993725	25.17	0.000^***^
p8	Catast	0.4874306	0.0423102	11.52	0.000^***^
	cons	1.579521	0.0733251	21.54	0.000^***^
p17	Catast	0.7251901	0.0296025	24.50	0.000^***^
	cons	2.100107	0.0875678	23.98	0.000^***^
p26	Catast	0.5695476	0.0387881	14.68	0.000^***^
	cons	1.702055	0.0765352	22.24	0.000^***^
p35	Catast	0.8137767	0.0253621	32.09	0.000^***^
	Cons	2.000841	0.0847412	23.61	0.000^***^
p9	Anoth-blame	0.8759206	0132696	66.01	0.000^***^
	cons	1.941513	0.0830745	23.37	0.000^***^
p18	Anoth-blame	0.9647114	0.0085807	112.43	0.000^***^
	cons	1.929159	0.0827291	23.32	0.000^***^
p27	Anoth-blame	0.6590473	0.0291331	22.62	0.000^***^
	cons	2.238683	0.0915817	24.44	0.000^***^
p36	Anoth-blame	0.8563878	0.0152979	55.98	0.000^***^
	cons	2.238683	0.0768105	22.29	0.000^***^

* *p < 0.05*,

** *p < 0.01*,

*** *p < 0.001*.

In relation to model fit, we will consider a set of fit measures to conveniently confirm the resulting factor structure. First, we consider the absolute fit indices, i.e., the likelihood ratio, the root mean squared error of approximation (RMSEA), as well as the standardised root mean squared residual (SRMR). For the likelihood ratio, and given the violation of the multinormality assumption, we have considered the calculation of the S-B scaled test χ^2^ = 1069.08; df = 558; *p* < 0.001 (Santorra and Bentler, 1990) to be more appropriate. The result obtained shows that the empirical model obtained does not fit the theoretical one. Evidently, the occurrence of this misfit is usual when there is a breach of multivariate normality due to the maximisation of the χ^2^ values when estimated by the ML method. As for the RMSEA = 0.0498 (90% CI lower bound = 0.046; upper = 0.054) and the SRMR = 0.0620. If we take into account the indications of Bollen and Long (1993) and Hu and Bentler (1999) that values of <0.05 for RMSEA and <0.08 for SRMR indicate a good fit, it seems that in our case we have obtained a remarkably adjusted model. Second, we considered two measures of comparative fit, namely the Tucker-Lewis index (TLI) and the comparative fit index (CFI). The TLI value = 0.90 and the CFI = 0.91. With Widaman and Thompson (2003) and Yuan (2005) we can state that, since values <0.90 indicate questionable fits, there are sufficient empirical reasons to claim that the model in our study has achieved a good fit.

However, the excessive presence of female vs. male gender in the sample under study generates an evident imbalance that could bias the results obtained in the validation of the CERQ scale (Byrne, 2008). For this reason, we have subjected the scale to factorial measurement by gender. With González-Betanzos et al. (2015, p. 29) we must remember that measurement invariance (MI) implies that the measurement properties of the scale or of the items of a scale should be independent of the distinctive characteristics of the people who have completed it, except for the specific characteristics that are being measured with that scale.

Regarding the invariance analysis of the factor structure, the fit indices obtained (Table 6) allow us to accept the equivalence of the basic measurement models between the two samples, i.e., between males vs. females, since, on the one hand, they are very similar and, on the other hand, they are within the appropriate limits to consider them separately as two confirmatory factor analyses suitably adjusted.

**Table 6:** Fit measures of confirmatory factor analysis (CFA) females vs. males.

	**Fit measures**				**RMSEA 90% CI**	
**Gender**	**CFI**	**TLI**	**SRMR**	**RMSEA**	**Lower**	**Upper**
Males	0.837	0.816	0.0849	0.0730	0.0618	0.0836
Females	0.886	0.872	0.0718	0.0559	0.0503	0.0614

*CFI, comparative fit index; TLI, Tucker-Lewis index; RMSEA, root mean square error of approximation*.

## Discussion and Conclusions of Study

The main contribution of the present study is that it is the first time that the psychometric properties of the CERQ-S36 scale have been tested in a Spanish University sample made up of students from different degree programs. To this consideration we must add that the scale was administered to these students during the Covid-19 confinement, a situation of unprecedented health, social and economic emergency at national and global level, ideal for testing the difficulties of emotion regulation in the sample of students under investigation, as well as the stability and consistency of the scale. As for the psychometric results of the scale and taking into consideration the different typologies, reliability and validity from the Classical Test Theory (CTT) were as follows.

Firstly, in relation to reliability as internal consistency, we note that the scale used with University students in a Covid-19 scenario has obtained value of McDonald's ω coefficient of 0.83. These results, that confirm the high stability of the scale in a global emergency situation, are in line with the results of reliability as internal consistency obtained by the CERQ-S36 scale in its Spanish version for different age groups, both in the Spanish context (Feliu-Soler et al., 2017; Orgilés et al., 2018; Chamizo-Nieto et al., 2020), and in the Latin American context (Medrano et al., 2013; Domínguez and Medrano, 2016). At the international level, we have also found similar results to our study in English-language papers, for example, those of Kraaij and Garnefski (2019), Ireland et al. (2017), and Tuna and Bozo (2012), whose reliability as internal consistency has reached moderately high values (similar to our study). All these results constitute empirical evidence, without a doubt, of the high level of stability and consistency of the CERQ scale accredited at a general level and as a reference instrument when measuring the difficulties of emotion regulation, even in periods of emergency and high-impact crisis situations.

Secondly, with reference to concurrent criterion validity, we can conclude that the items individually measure the same as the total scale (internal criterion), given that the item-rest correlation values are generally above *r* > 0.35. Obviously, we cannot appreciate any kind of predictive criterion validity, since we have not been able to compare the overall results of the CERQ-S36 scale with any other scale measuring relevant constructs for such a comparison.

Thirdly, in relation to construct validation we have calculated two factor analyses; one of an exploratory nature and the other of a confirmatory nature. The first exploratory factor analysis resulted in a factor structure identical to that obtained by the original scale of Garnefski et al. (2002a). In this way, we have confirmed a structure built around the nine original dimensions of the aforementioned authors. The values obtained for the statistics used for the assumption cheques of adequacy were all satisfactory, ruling out the presence of unsuitable matrices for their calculation (identity and singular matrices) and, on the contrary, the presence of satisfactory measures of overall and individual sample adequacy. As for the results of the exploratory factor analysis, we highlight the moderate percentage of variance explained by the inferred factor structure, as well as the moderate values obtained for the communality of each variable in this factor solution, which denote its good representation.

The second factor analysis, of a confirmatory nature, served precisely to confirm the factor structure obtained previously by the exploratory analysis. In the resulting path analysis, it can be seen that a factor solution has been achieved based on the nine original factors of the scale. In general, the regression weights of each empirical variable considered, in relation to each of the latent factors of which it is a part, have obtained values of *r* > 0.40. However, there was one case, that of item 20 (I think I can't change it), which obtained a regression weight of *r* < 0.40, exactly a value of *r* = 0.16. This incidence has already been reported in some previous works (Jermann et al., 2006; Domínguez-Sánchez et al., 2011; Tuna and Bozo, 2012; Medrano et al., 2013; Ireland et al., 2017 or more recently in the work of Chamizo-Nieto et al., 2020). Chamizo-Nieto et al. (2020) indicate that, although this item belongs to the “Acceptance” factor, it seems to be closer to resignation as a passive rather than an active process, suggesting that further research is needed on this incidence, which has been described in other works that study different populations and are written in different languages. As for the measures of fit, both the absolute and comparative fit indices, we have obtained more than enough empirical evidence to be able to affirm that the inferred empirical model has obtained a consistent fit in relation to the theoretical starting model.

In short, the results obtained suggest that the CERQ-S36 scale could be useful for assessing cognitive coping in University populations in times of crisis, such as the current global emergency caused by the presence of Covid-19. This would allow, as Chamizo-Nieto et al. (2020) indicate, to expand the study and knowledge of emotional regulation strategies, how these emotions influence and what consequences they have on the health and psychological functioning of University students in crisis situations.

## Data Availability Statement

The original contributions presented in the study are included in the article/supplementary material, further inquiries can be directed to the corresponding author.

## Ethics Statement

Ethical review and approval was not required for the study on human participants in accordance with the local legislation and institutional requirements. Written informed consent was not provided because it is a totally anonymized investigation, where only opinions are requested.

## Author Contributions

CR-S analysed and interpreted the data. PI-C, JÁ-R, and SL-R contributed reagents, materials, and analysis tools or data. All authors listed have significantly contributed to the development and the writing of this article and conceived and designed the paper.

## Conflict of Interest

The authors declare that the research was conducted in the absence of any commercial or financial relationships that could be construed as a potential conflict of interest.

## Footnotes

^1^From now on.
